# Chronic sustained hypoxia-induced redox remodeling causes contractile dysfunction in mouse sternohyoid muscle

**DOI:** 10.3389/fphys.2015.00122

**Published:** 2015-04-20

**Authors:** Philip Lewis, David Sheehan, Renata Soares, Ana Varela Coelho, Ken D. O'Halloran

**Affiliations:** ^1^Department of Physiology, School of Medicine, University College CorkCork, Ireland; ^2^School of Biochemistry and Cell Biology, University College CorkCork, Ireland; ^3^Instituto de Tecnologia Química e Biológica António Xavier, New University of LisbonLisbon, Portugal

**Keywords:** hypoxia, oxidative stress, antioxidants, respiratory muscle, COPD

## Abstract

Chronic sustained hypoxia (CH) induces structural and functional adaptations in respiratory muscles of animal models, however the underlying molecular mechanisms are unclear. This study explores the putative role of CH-induced redox remodeling in a translational mouse model, with a focus on the sternohyoid—a representative upper airway dilator muscle involved in the control of pharyngeal airway caliber. We hypothesized that exposure to CH induces redox disturbance in mouse sternohyoid muscle in a time-dependent manner affecting metabolic capacity and contractile performance. C57Bl6/J mice were exposed to normoxia or normobaric CH (FiO_2_ = 0.1) for 1, 3, or 6 weeks. A second cohort of animals was exposed to CH for 6 weeks with and without antioxidant supplementation (tempol or N-acetyl cysteine in the drinking water). Following CH exposure, we performed 2D redox proteomics with mass spectrometry, metabolic enzyme activity assays, and cell-signaling assays. Additionally, we assessed isotonic contractile and endurance properties *ex vivo*. Temporal changes in protein oxidation and glycolytic enzyme activities were observed. Redox modulation of sternohyoid muscle proteins key to contraction, metabolism and cellular homeostasis was identified. There was no change in redox-sensitive proteasome activity or HIF-1α content, but CH decreased phospho-JNK content independent of antioxidant supplementation. CH was detrimental to sternohyoid force- and power-generating capacity and this was prevented by chronic antioxidant supplementation. We conclude that CH causes upper airway dilator muscle dysfunction due to redox modulation of proteins key to function and homeostasis. Such changes could serve to further disrupt respiratory homeostasis in diseases characterized by CH such as chronic obstructive pulmonary disease. Antioxidants may have potential use as an adjunctive therapy in hypoxic respiratory disease.

## Introduction

The striated muscles of breathing are the final common effectors of the respiratory control system, critical in the maintenance of respiratory homeostasis. Pharyngeal dilator muscles are an important subset of the respiratory muscles serving to control the patency of the upper airway. Pharyngeal dilator muscle damage or dysfunction increases the risk of obstructive airway events. Upper airway muscle dysfunction is implicated in human obstructive sleep apnea (OSA), a common respiratory disorder characterized by repeated occlusions of the pharyngeal airway during sleep. Of note, OSA is prevalent in patients with chronic obstructive pulmonary disease (COPD), presenting as the “overlap syndrome” (Owens et al., 2008; McNicholas, 2009; Owens and Malhotra, 2010), but the reasons for the emergence of this co-morbidity are unclear.

Chronic sustained hypoxia (CH) is a dominant feature of respiratory diseases including COPD, but the putative role of CH in respiratory muscle remodeling and dysfunction is generally under-explored. Little is known about the effects of CH exposure on the respiratory muscles and there exists a general paucity of information concerning the pharyngeal dilator muscles despite the potential clinical relevance. Respiratory and limb muscle remodeling are features of COPD (Orozco-Levi, 2003; Doucet et al., 2004, 2010; Levine et al., 2013) and interestingly, exposure to CH elicits differential structural and functional adaptations in respiratory and limb muscles (El-Khoury et al., 2003, 2012; Faucher et al., 2005; McMorrow et al., 2011; Gamboa and Andrade, 2012; Carberry et al., 2014). We speculate that hypoxic remodeling of respiratory muscle has relevance to respiratory muscle plasticity in respiratory disease. Hypoxia-related remodeling of upper airway muscles may underpin poor upper airway control in COPD.

Pharyngeal dilator muscles are phasically active during inspiration and muscle activity is increased during hypoxic exposure (O'Halloran et al., 2002; Edge et al., 2014). Contractile activity increases reactive oxygen species (ROS) production which are pivotal for muscle adaptation to high altitude and respiratory diseases characterized by hypoxia (Zuo and Clanton, 2005; Marin-Corral et al., 2009; Murray, 2009; Chaudhary et al., 2012; El-Khoury et al., 2012; Levine et al., 2013; Puig-Vilanova et al., 2015). Thus, CH likely challenges respiratory muscles, including the pharyngeal dilator muscles, with enhanced ROS on two fronts—increased contractile activity during reflex hyperventilation and hypoxia *per se*. We postulated a role for ROS in eliciting CH-induced redox remodeling of key muscle proteins with resultant functional changes in the sternohyoid muscle—a representative pharyngeal dilator muscle. The supra- and infra-hyoid muscles of the upper airway control the position of the hyoid bone, displacing it anteriorly during inspiration to enlarge the caliber of the pharynx. Sternohyoid muscle length correlates to airway volume (Van Lunteren et al., 1987) and the muscle is recruited during increased respiratory drive (O'Halloran et al., 2002) and obstructive airway events (Edge et al., 2012). Sternohyoid muscle damage is reported in the English bulldog, an animal model of OSA (Petrof et al., 1994). We have extensively characterized sternohyoid muscle physiology (O'Halloran, 2006; Skelly et al., 2010; Shortt and O'Halloran, 2014; McDonald et al., 2015), including several studies in animal models of chronic hypoxia (Bradford et al., 2005; McMorrow et al., 2011; Skelly et al., 2011, 2012a,b, 2013; O'Connell et al., 2013; Carberry et al., 2014; McDonald et al., 2014). The sternohyoid muscle is suited to *ex vivo* study of contractile function due to ease of access, longitudinal arrangement of its fibers and the opportunity, employed in the present study, to keep bony origin and insertions intact in isolated preparations.

Given that ventilation, muscle contractile behavior, and the level of hypoxia experienced by the respiratory muscles, are likely temporally modified during the process of acclimatization to CH, we characterized protein carbonyl and free thiol content (the most common and most specific form of protein oxidation respectively (Dalle-Donne et al., 2006; El-Shafey et al., 2011) in the sternohyoid after 1, 3, or 6 weeks of CH exposure. In addition, we sought to identify redox-modified proteins in the sternohyoid muscle after 6 weeks of CH using 2D redox proteomics combined with mass spectrometry (Cole et al., 2014; Hu et al., 2014; Rainville et al., 2014) having reasoned that this information will aid the determination of how and where ROS exert their effects in muscle cells following CH exposure. We postulated that chymotrypsin-like activity of the 20S proteasome is increased in the sternohyoid after 6 weeks of CH given that proteasomal activity is highly sensitive to ROS (McClung et al., 2008; Aiken et al., 2011). Hypoxia through the hypoxia-inducible factor (HIF)-1α transcription factor promotes a more glycolytic phenotype in tissues to reduce the reliance on oxygen in ATP production (Howald et al., 1990; Murray, 2009; Wheaton and Chandel, 2011). HIF expression correlates with muscle fiber type and activity and is also modulated by ROS. Therefore, we reasoned that glyceraldehyde-3-phosphate dehydrogenase (GAPDH) and lactate dehydrogenase (LDH) activities would be temporally modified in the sternohyoid in response to CH exposure. We also assessed HIF-1α content, phospho-MAPK content (p38, JNK, ERK1/2), and sternohyoid isotonic contractile and endurance properties *ex vivo* after 6 weeks of CH in the presence or absence of chronic antioxidant supplementation with tempol or N-acetyl cysteine (NAC). We tested the hypothesis that CH causes aberrant redox modulation of sternohyoid muscle function which is reversible by antioxidant supplementation.

## Materials and methods

### Ethical approval

All protocols involving animals described in this study were approved by local ethics committee and were performed under license from the Irish Government Department of Health and Children in accordance with EU legislation.

### Animal model

In the first series of experiments, 48 adult male C576Bl/J mice (Charles River Laboratories, UK) were exposed to 1, 3, or 6 weeks of CH (FiO_2_ = 0.1) or normoxia (6 groups: *n* = 8 per group, matched for age and weight) in environmental chambers (OxyCycler Model A84, BioSpherix Ltd, USA) with precise control of ambient oxygen concentration. All mice were housed at room temperature on a 12:12-h light–dark cycle. Food and water were available *ad libitum*, and the chambers were opened briefly once a week for cleaning. In a second series, 32 adult male C576Bl/J mice were exposed to normoxia or CH with or without antioxidant supplementation with tempol or NAC for 6 weeks (4 groups: *n* = 8 per group, matched for age and weight). At the end of the gas treatment periods, animals were anesthetized by 5% isoflurane inhalation in oxygen and euthanized by cervical dislocation. Blood samples were taken in capillary tubes for hematocrit determination.

### Molecular studies

#### Tissue preparation for molecular studies

Sternohyoid muscles were excised, snap frozen in liquid nitrogen and stored at −80°C. Frozen muscle samples were homogenized in 10% w/v modified radioimmunoprecipitation (RIPA) assay buffer (1X RIPA, 200 mM sodium fluoride, 1 mM phenylmethylsulfonyl fluoride, protease inhibitor cocktail, phosphatase inhibitor cocktail (Fisher Scientific, Ireland) and centrifuged at 13,000 *g* for 15 min to separate the insoluble fraction from crude protein homogenate. Protein concentrations were evaluated using a bicinchoninic assay (Pierce Biotechnology (Fisher Scientific), Ireland) against bovine serum albumin standards. Two additional muscles, the soleus and extensor digitorum longus (EDL), slow and fast fiber type limb muscles respectively, were also prepared in this manner for determination of total protein carbonyl and free thiol content for comparison to sternohyoid.

#### Total protein carbonyl and free thiol content

As previously described (Cole et al., 2014; Hu et al., 2014; Rainville et al., 2014), muscle homogenates were incubated with either 2 mM fluorescein-thiosemicarbazide (FTSC) or 2 mM iodoacetamidofluorescein (IAF) (Sigma-Aldrich Co., Ireland) for 2 h in the dark on ice for detection of free protein carbonyl and thiol groups respectively. Samples were then precipitated with 20% trichloroacetic acid (TCA) in acetone, followed by centrifugation at 11,000 *g* for 3 min. Protein pellets were then washed with ice-cold excess 1:1 ethylacetate/ethanol or acetone (for FTSC and IAF respectively) to remove excess TCA, interfering salts and non-protein contaminants. Samples were dried, re-suspended in sample buffer containing 5% beta-mercaptoethanol and heated at 95°C for 5 min before electrophoretic separation on a 12% polyacrylamide gel (1D). Fluorescent images of the gels were captured on a Typhoon Trio+ Variable-Mode Imager (GE Healthcare, UK). Protein bands were visualized by colloidal coomassie staining (Dyballa and Metzger, 2009) and images were captured on a calibrating image densitometer (GS-800, Bio-Rad, USA).

#### 2D redox proteomics

As previously described (Cole et al., 2014; Hu et al., 2014; Rainville et al., 2014), this method separates proteins according to their isoelectric point and molecular mass such that they appear as spots when stained on polyacrylamide gels; protein spots can be analyzed independently. Briefly, sternohyoid muscle samples were treated as described above for 1D preparation until re-suspension in sample buffer. Samples were instead re-suspended in rehydration buffer (7 M urea, 2 M thiourea, 2% (w/v) 3-[(3-Cholamidopropyl)dimethylammonio]-1-propanesulfonate, 4% (v/v) ampholytes (Pharmalyte 3–10, Amersham, UK), 1% (v/v) destreak reagent (Amersham) and a trace amount of bromophenol blue) and loaded onto 70 mm pH 3–10 non-linear immobilized pH gradient (IPG) strips (GE Healthcare) overnight. IPG strips were focused on a Protean isoelectric focusing (IEF) cell (BioRad) with linear voltage increases: 250 V for 15min; 4000 V for 2 h; then up to 20,000 V. Following IEF, strips were equilibrated (20 min) in equilibration buffer (6 M urea, 0.375 M Tris, pH 8.8, 2% (w/v) sodium-dodecyl sulfate, and 20% (v/v) glycerol) containing 2% (w/v) dithiothreitol, and then for 20 min in equilibration buffer containing 2.5% (w/v) iodoacetamide. Equilibrated strips were then subjected to gel electrophoresis. Fluorescent and colloidal coomassie stained gel images were captured as described above.

#### Image analysis

Quantity One image analysis software (Bio-Rad) was used to subtract background and quantify optical density for total protein carbonyl and free thiol content. For each sample, intensity of fluorescence was normalized to intensity of coomassie staining. For 2D separations, alignment of gels, spot matching, and quantification of spot volumes was carried out using Progenesis SameSpots image analysis software (Version 4.5; Non-linear Dynamics, USA).

#### Protein digestion and identification

Gel spots were used for in-gel protein digestion with trypsin. The extracted peptides were loaded onto a R2 micro column (RP-C18 equivalent) where they were desalted, concentrated and eluted directly onto a MALDI plate using α-cyano-4-hydroxycinnamic acid (5 mg/ml) as matrix solution in 50% (v/v) acetonitrile and 5% (v/v) formic acid. Mass spectra of the peptides were acquired with positive reflectron mass spectrometry (MS) and MS/MS modes using MALDI-TOF/TOF MS instrument (4800 *plus* MALDI TOF/TOF analyzer). The collected MS and MS/MS spectra were analyzed in combined mode using Mascot (version 2.2; Matrix Science, Boston, MA) search engine and SwissProt (release 02_2013, 539,165 entries) database restricted to 50 ppm peptide mass tolerance for the parent ions, an error of 0.3 Da for the fragments, one missed cleavage in peptide masses, and carbamidomethylation of Cys and oxidation of Met as fixed and variable amino acid modifications, respectively. No taxonomy restrictions were applied. The identified proteins were only considered if a MASCOT score above 95% confidence was obtained (*p* < 0.05) and at least one peptide was identified with a score above 95% confidence (*p* < 0.05). This analysis was conducted by the Analytical Services Unit, Instituto de Tecnologia Química e Biológica (ITQB), New University of Lisbon, Lisbon, Portugal.

#### Chymotrypsin-like proteasome activity

Chymotrypsin-like activity of the 20S proteasome was measured fluorometrically in accordance with the manufacturer's instructions (Abcam, UK).

#### Glycolytic enzyme activities

For GAPDH activity measurement, samples were added to 13.5 mM sodium pyrophosphate buffer (pH 8.5) containing 30 mM sodium arsenate, 0.25 mM NAD with 3.325 mM DTT. Samples were incubated at 25°C for 10 min to achieve temperature equilibration and to establish a blank rate, if any. 0.5 mM DL-glyceraldehdye-3-phosphate was added and absorbance was recorded for 10 min at 339 nm. Measured rates were corrected by measuring the blank rate of the reaction. One unit is defined as 1 μmol NADH generated/minute/mg protein. Similarly, total LDH activity was calculated as rate of absorbance at 339 nm produced by oxidation of NADH at 25°C and pH 7.3 in 0.2 M Tris-HCL buffer containing 1 mM sodium pyruvate and 0.22 mM NADH.

#### HIF-1α and phospho-MAPK content

HIF-1α content and phospho-(p)-p38, p-JNK, p-ERK1/2 content were assayed by an immuno-linked luminescence assay in accordance with manufacturer's instructions (Mesoscale Discovery, Gaithersburg, USA). HeLa cells treated with and without cobalt chloride for 16 h provided positive and negative controls respectively for the HIF-1α assay. Lysate from Jurkat cells treated with 1 mol rapamycin for 3 h to activate MAPK phosphatase 1 was used as negative control for the MAPK assay. Lysate from Jurkat cells treated with 50 nM calyculin A and 200 nM PMA for 15 min to stimulate phosphorylation of MAPK proteins was used as a positive control (Mesoscale Discovery).

### Isotonic muscle function

#### *Ex vivo* muscle preparation

Animals were anesthetized by 5% isoflurane inhalation in air and euthanized by cervical dislocation. Sternohyoid muscles were excised and placed in a bath of Krebs solution (NaCl 120 mM, KCl 5 mM, Ca^2+^ gluconate 2.5 mM, MgSO_4_ 1.2 mM, NaH_2_PO_4_ 1.2 mM, NaHCO_3_ 25 mM, glucose 11.5 mM, and 25 μM d-tubocurarine) at room temperature and gassed with carbogen (95% O_2_/5% CO_2_) before mounting in the test bath for assessment of muscle contractile and endurance performance. Whole intact mouse sternohyoid muscle bundles were arranged between the electrodes with the sternum anchored at a fixed base and hyoid bone connected by a non-elastic string to a dual-mode lever force transducer (Aurora Scientific, Canada) such that fibers were orientated vertically. Upon placement in the test bath, bundles were incubated at 35°C in Krebs solution gassed with carbogen (95% O_2_/5% CO_2_). Muscle performance has previously been shown (Skelly et al., 2010) to be optimal under hyperoxic conditions (95% O_2_) compared to normoxic conditions (21% O_2_). Bundles were equilibrated for 5 min in gassed Krebs solution prior to initiating the experimental protocol.

#### Protocol

**Figure 1:**
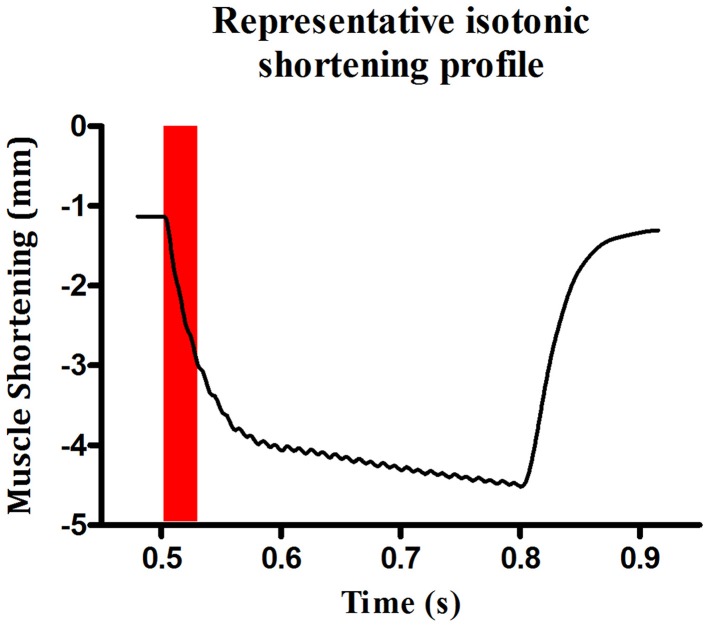
**Representative profile of sternohyoid peak shortening velocity**. An original record illustrating a representative contraction at 0% load. Maximum velocity of shortening was calculated during the first 30 ms of the contraction.

Bundles were set to optimum length (Lo—length at which peak twitch force occurs) by adjusting the position of the force transducer with a micro-positioner and stimulating with repeated single pulses whilst adjusting muscle length until peak twitch force was reached. Contractile kinetics [time to peak (TTP) and half-relaxation time (T50)] were measured from the peak twitch force recordings. After 5 min of equilibration, the lever of the force transducer was set to maximum rigidity (~500 mN; >100% load) and a tetanic contraction was elicited by stimulating the bundle with supra-maximal voltage at 100 Hz for 300 ms. The peak isometric force (F*max*) was established allowing determination of 100% load for isotonic assessments. Shortening contractions were elicited in incremental steps ranging from 0 to 100% load with 1 min rest between each step. Peak shortening velocity (V*max*) was determined during the initial 30 ms of shortening (see Figure 1) as this is when velocity is greatest (Watchko et al., 1997; Van Lunteren et al., 2007; Van Lunteren and Pollarine, 2010). Power was determined at each step as the product of force/cross-sectional area (CSA) *x* shortening velocity/Lo. Peak specific work (W*max*) was calculated as force/CSA *x* shortening (L/Lo) at the load where the product of these variables was greatest.

#### Isotonic function data analysis

Muscle cross-sectional area (CSA) was calculated as the blotted dry muscle bundle weight divided by the product of Lo and the specific density which was assumed to be 1.056 g/cm^3^. Specific force was calculated as N/cm^2^. Specific shortening velocity was calculated as Lo/s. Specific power was calculated as Watts/cm^2^. W*max* was calculated as Joules/cm^3^.

#### Statistical analyses

Statistical comparisons of the total mean fluorescence intensity for 1D preparations and FTSC/IAF- and coomassie-labeled spot volumes (2D) were measured using Student *t*-tests, Mann-Whitney *U*-tests, or One-Way ANOVA as appropriate after testing for normality and equal variance in the data sets. Student *t*-test or Mann-Whitney *U*-test was used as appropriate to compare control and CH groups for chymotrypsin-like activity of the 20S proteasome, and GAPDH and LDH activities. Tissue limitations restricted the measurement of all molecular components at all three time points of CH exposure. HIF-1α content and p-MAPK contents were compared between groups by One-Way ANOVA followed by Tukey's multiple comparison *post-hoc* test, or the Kruskal-Wallis test followed by Dunn's multiple comparison *post-hoc* test as appropriate, after testing for normality and equal variance in the data sets. Statistical comparisons were performed between groups for sternohyoid contractile and endurance performance using One-Way ANOVA followed by Tukey's multiple comparison *post-hoc* tests. Power-load relationship was assessed by Two-Way ANOVA followed by Bonferroni's *post-hoc* multiple comparison tests. Linear regression analysis was performed to assess the relationship between oxidative stress markers (protein free thiol and carbonyl groups) and sternohyoid muscle power. *P* < 0.05 was the criterion for statistical significance in all tests.

## Results

### Animal model

Hematocrit was significantly (*p* < 0.001) increased by CH exposure (Table 1). Antioxidant supplementation in animals exposed to 6 weeks of CH had no effect on hematocrit compared to CH alone. Animals drank equivalent volumes of water (with or without antioxidant) per day. Body mass decreased after 1 week of CH exposure but all hypoxic animals returned to a growth curve equivalent to the control animals for weeks two through to six of the gas treatments (data not shown).

**Table 1:** **Hematocrit values after 1, 3, or 6 weeks of normoxia or chronic sustained hypoxia (CH)**.

	**Normoxia**	**CH**	***P*-value^a^**
**HEMATOCRIT (%)**			
One Week	34 ± 2	52 ± 2	*P* < 0.001
Three Weeks	38 ± 1	65 ± 1	*P* < 0.001
Six Weeks	32 ± 1	62 ± 1	*P* < 0.001

*Data are presented as mean ± SEM; n = 8 per group*.

a *Student unpaired t-test*.

### Total protein carbonyl and free thiol content

**Figure 2:**
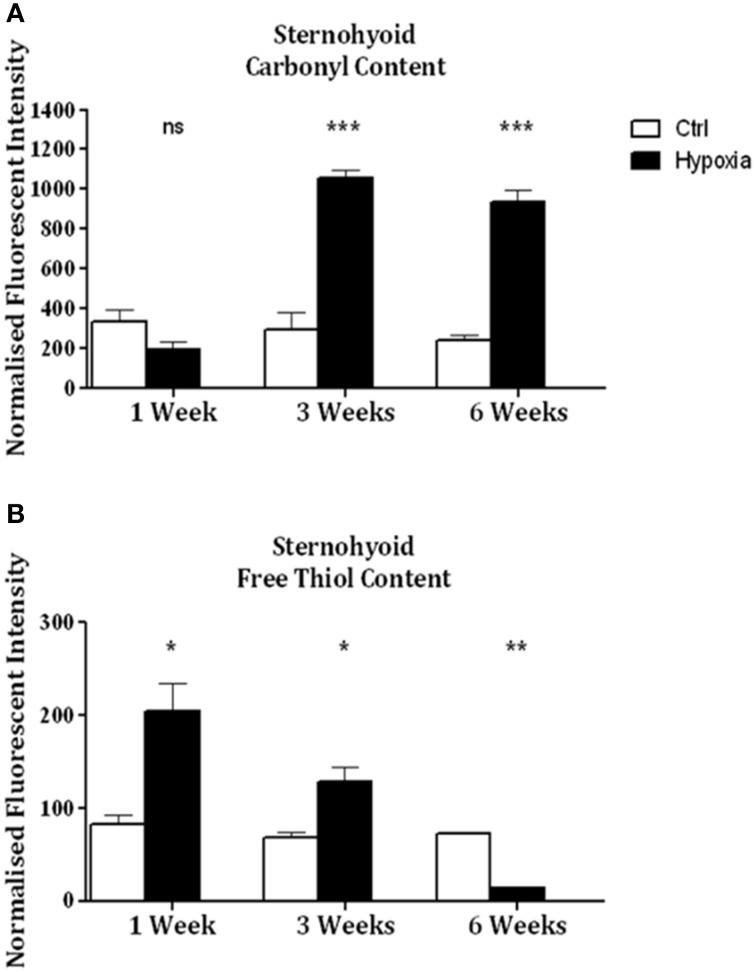
**Sternohyoid protein carbonyl and free thiol content after 1, 3, and 6 weeks of sustained hypoxia compared to normoxic controls. (A)** Sternohyoid protein carbonyl content (mean ± SEM) after 1, 3, and 6 weeks of normoxia or sustained hypoxia expressed as normalized fluorescence intensity; *n* = 5–8 per group; **(B)** Sternohyoid protein free thiol content (mean ± SEM) after 1, 3, and 6 weeks of normoxia or sustained hypoxia expressed as normalized fluorescence intensity; *n* = 7–8 per group; ^*^p < 0.05, ^**^p < 0.01, ^***^p < 0.001, ns, not significant; Student *t*-test or Mann-Whitney test as appropriate. Ctrl, normoxic control; Hypoxia, sustained hypoxia (FiO_2_ = 0.1).

**Figure 3:**
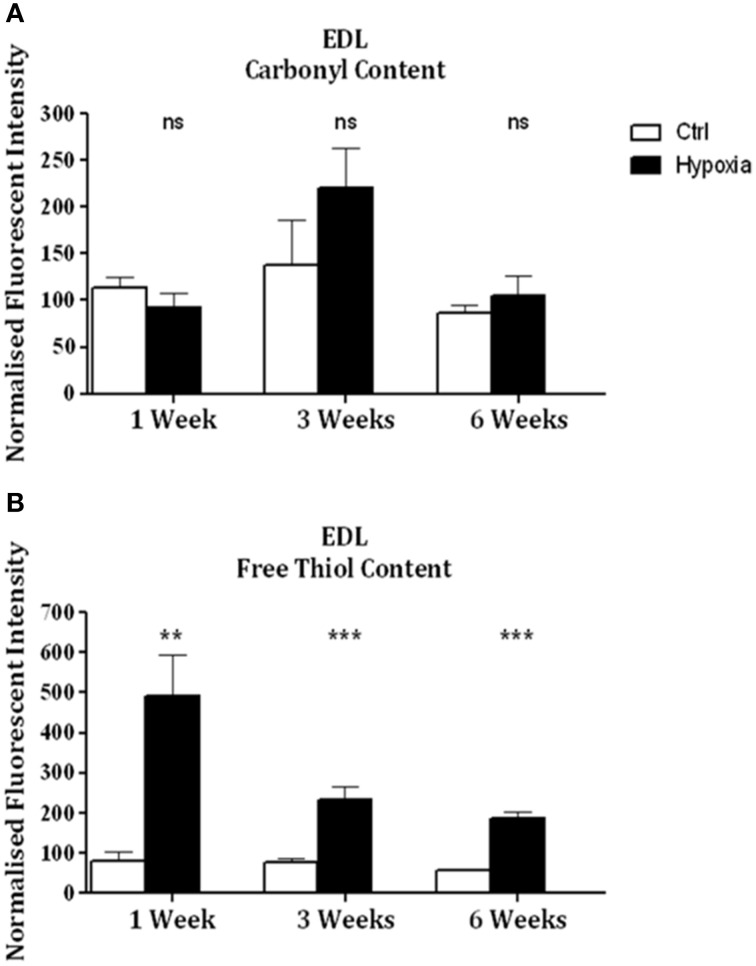
**EDL protein carbonyl and free thiol content after 1, 3, and 6 weeks of sustained hypoxia compared to normoxic controls. (A)** EDL protein carbonyl content (mean ± SEM) after 1, 3, and 6 weeks of sustained hypoxia expressed as normalized fluorescence intensity; *n* = 4–8 per group; **(B)** EDL protein free thiol content (mean ± SEM) after 1, 3, and 6 weeks of sustained hypoxia expressed as normalized fluorescence intensity; *n* = 6–8 per group; ^**^p < 0.01, ^***^p < 0.001, ns, not significant; Student *t*-test or Mann-Whitney test as appropriate; Ctrl, normoxic control; Hypoxia, sustained hypoxia (FiO_2_ = 0.1).

**Figure 4:**
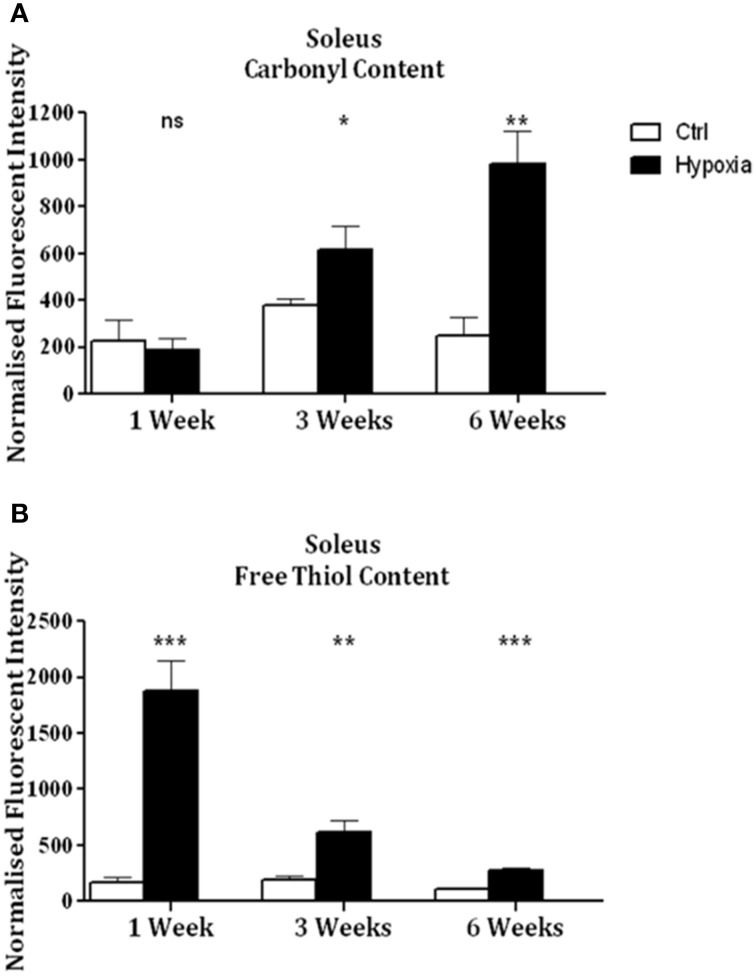
**Soleus protein carbonyl and free thiol content after 1, 3, and 6 weeks of sustained hypoxia compared to normoxic controls. (A)** Soleus protein carbonyl content (mean ± SEM) after 1, 3, and 6 weeks of sustained hypoxia expressed as normalized fluorescence intensity; *n* = 4–8 per group; **(B)** Soleus protein free thiol content (mean ± SEM) after 1, 3, and 6 weeks of sustained hypoxia expressed as normalized fluorescence intensity; *n* = 8 per group; ^*^p < 0.05, ^**^p < 0.01, ^***^p < 0.001, ns, not significant; Student *t*-test or Mann-Whitney test as appropriate; Ctrl, normoxic control; Hypoxia, sustained hypoxia (FiO_2_ = 0.1).

Significant increases in sternohyoid carbonyl content were observed after 3 (*p* < 0.001) and 6 (*p* < 0.001) weeks of CH (Figure 2A)—indicative of increased sternohyoid protein oxidation. Sternohyoid protein free thiol content changes were bi-phasic (Figure 2B). There was a significant increase in sternohyoid protein free thiol content after 1 (*p* < 0.05) and 3 (*p* < 0.01) weeks of CH; however free thiol content was significantly lower than control after 6 weeks of CH (*p* < 0.001). For comparison, two limb muscles, namely soleus, and extensor digitorum longus (EDL) were also studied. Unlike the sternohyoid, there were no significant changes to the carbonyl content of the EDL proteome after CH exposure (Figure 3A). There was, however, significant increases in EDL free thiol content after 1 (*p* < 0.01), 3 (*p* < 0.001), and 6 (*p* < 0.001) weeks of CH—though the magnitude of the increase is seen to decrease over time (Figure 3B). Similar to the sternohyoid muscle, but unlike the EDL, significant increases in soleus protein carbonyl content were observed after 3 (*p* < 0.05), and 6 (*p* < 0.01) weeks of CH (Figure 4A). Soleus protein free thiol content was significantly increased after 1 (*p* < 0.001), 3 (*p* < 0.01), and 6 (*p* < 0.001) weeks of CH compared to controls (Figure 4B). Consistent with EDL, there was a decline in increased free thiol content in the soleus with progressive CH.

### 2D redox proteomics

**Figure 5:**
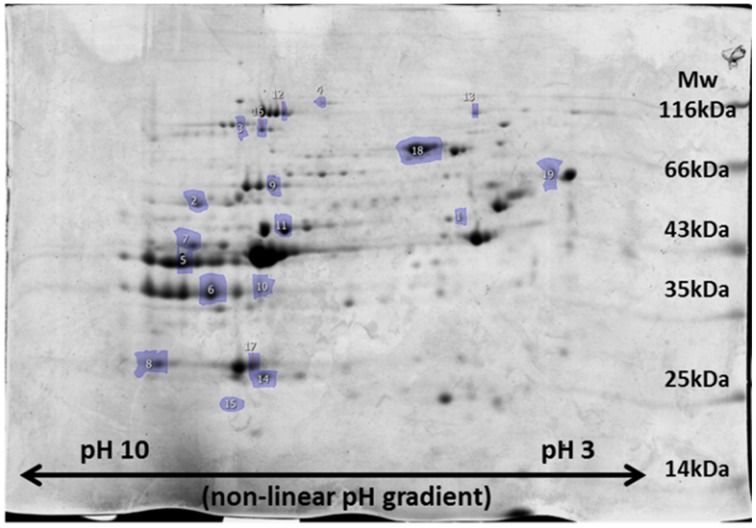
**Representative image of 2D-PAGE sternohyoid muscle protein profile after coomassie staining of proteins**. A molecular mass marker ranging from 14 to 116 kDa is shown for size reference and isoelectric point is indicated along the range pH 10-3. Spot numbers match those presented in Table 2.

In the sternohyoid, 498, 264, and 996 resolved spots were matched in FTSC-, IAF-labeled and coomassie-stained separations respectively. A significant relative volume difference was observed in 87 FTSC, 51 IAF, and 156 coomassie spots comparing control and CH groups (*p* < 0.05). Proteins were selected for mass spectrometry analysis based on separation, resolution, abundance, and overlap in fluorescence-stain and muscle separations. Protein smears and gel defects were excluded. Results for protein remodeling are presented in Table 2 with proteins grouped according to cellular location and/or function. Selected spots can be visualized on the representative coomassie stained gel shown in Figure 5. A change in FTSC and IAF fluorescence intensity signal independent of, or differential to, coomassie signal is indicative of protein redox remodeling.

**Table 2:** **Identifications by mass spectrometry of proteins undergoing significant redox remodeling in the sternohyoid muscle after 6 weeks of sustained hypoxia**.

**Spot #**	**Protein**	**Mw (Da)**	**GI number**	**Mascot score**	**Matched peptides**	**Sequence coverage**	**Carbonyl *p*-value, fold**	**Free thiol *p*-value, fold**	**Expression *p*-value, fold**
**ELECTRON TRANSPORT CHAIN**									
1	Cytochrome bc - 1 complex subunit 1	52,819	341941780	430	6	26%	-	<0.01, −1.4	<0.001, −2
2	ATP Synthase subunit α	59,716	416677	997	6	53%	-	-	<0.01, +1.5
**TCA CYCLE**									
3	Aconitase hydratase	85,410	60391212	1210	11	41%	<0.01, +1.4	<0.001, −2	<0.01, −2.6
4	2-oxo-glutarate dehydrogenase, mt	116,375	146345472	477	4	22%	-	<0.01, −2.4	<0.01, +2.8
**GLYCOLYSIS**									
5	Fructose bis-phosphate aldolase A	39,331	113607	588	3	70%	-	<0.01, −2.9	-
6	Glyceraldehyde-3-phosphate dehydrogenase	35,787	120702	531	4	48%	-	<0.01, −1.6	<0.001, −2
7	Phosphoglycerate kinase 1	44,534	1730519	534	4	45%	-	-	<0.01, +1.7
8	Phosphoglycerate mutase 2	28,809	6093745	1010	9	57%	-	-	<0.01, +1.7
9	Pyruvate kinase isozymes M1/M2	57,305	146345448	837	6	53%	<0.01, −1.2	-	<0.01, +1.3
**PHOSPHAGEN AND LIPID METABOLISM**									
10	Glycerol-3-phosphate dehydrogenase [NAD(+)]	37,548	121557	412	3	48%	-	<0.01, −2	<0.01, −2.6
11	Creatine kinase m-type	43,014	124056470	784	7	42%	<0.01, +1.2	-	<0.01, −1.2
**STRESS RESPONSE AND IRON HOMEOSTASIS**									
12	Glycogen phosphorylase	97,225	14916635	1450	13	47%	<0.01, −1.3	-	<0.01, +4.1
13	90kDa heat shock protein	83,229	341941065	742	6	31%	-	-	<0.01, −1.3
14	Alpha crystallin B chain	20,056	6166129	761	7	65%	<0.01, +1.2	-	-
15	Myoglobin	17,059	127676	923	7	43%	-	-	<0.01, −1.7
16	Serotransferrin	76,674	21363012	121	1	17%	<0.01, −1.4	<0.01, +1.8	-
17	Carbonic anhydrase III	29,348	30581036	966	8	71%	<0.01, +1.2	<0.01, −2.7	<0.01, +1.9
18	Albumin	68,648	5915682	1340	11	53%	-	-	<0.01, −1.8
**CROSS-BRIDGE AND SARCOPLASMIC RETICULUM**									
19	Calsequestrin	46,420	341940315	543	4	19%	<0.01, −1.5	-	<0.01, −2.3

### Sternohyoid glycolytic enzyme activities

Sternohyoid GAPDH activity was significantly increased after 1 week of CH (*p* < 0.05) but significantly decreased after 3 weeks of CH (*p* < 0.01) (data not shown). LDH activity in the sternohyoid (data not shown) was significantly increased after 1 week of CH (*p* < 0.05).

### Sternohyoid chymotrypsin-like proteasome activity

Chymotrypsin-like proteasome activity was unchanged in the sternohyoid after 6 weeks of CH (data not shown).

### Sternohyoid HIF-1α content

There was no change in sternohyoid HIF-1α content following 6 weeks of CH with or without antioxidant supplementation (data not shown).

### Sternohyoid phospho-MAPK content

**Figure 6:**
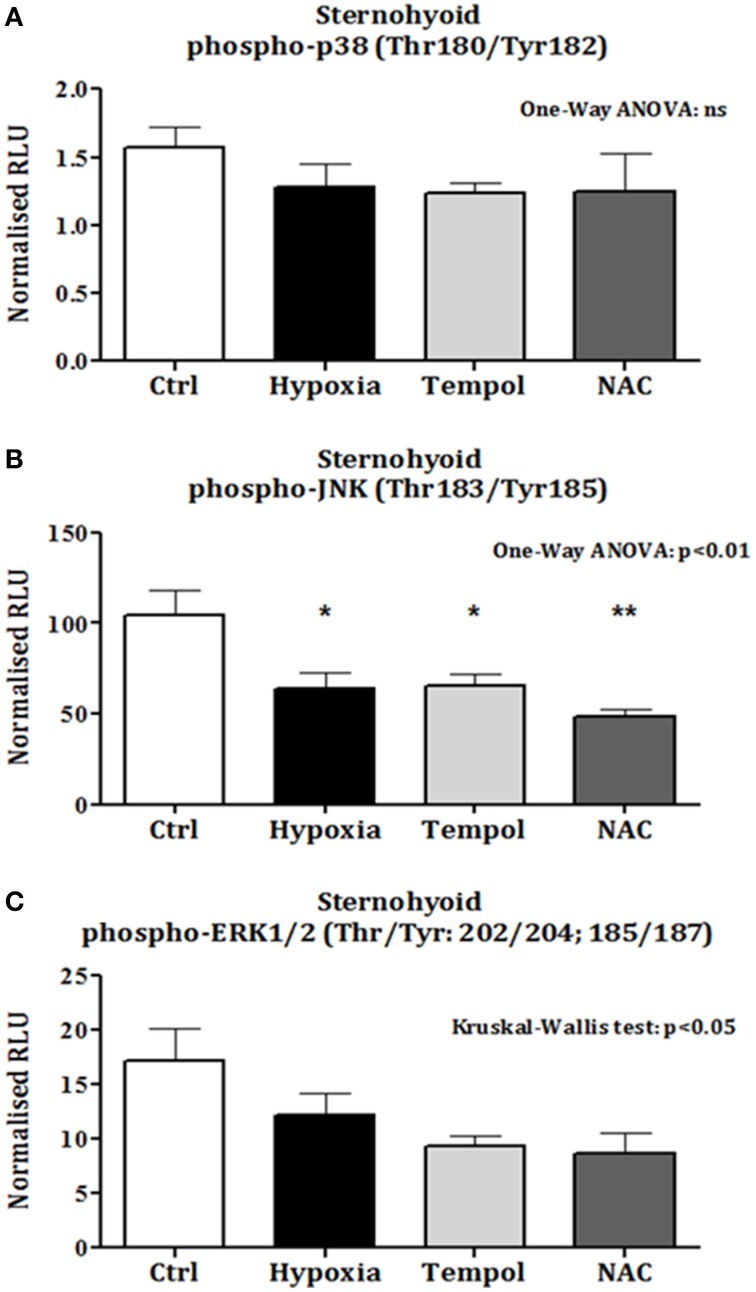
**Sternohyoid phospho-MAPK contents after 6 weeks of normoxia and sustained hypoxia ± chronic antioxidant supplementation. (A)** Sternohyoid phospho-p38 content (mean ± SEM) expressed as normalized relative luminescence units; *n* = 5–7 per group; **(B)** Sternohyoid phospho-JNK content (mean ± SEM expressed as normalized relative luminescence units; *n* = 5–7 per group; **(C)** Sternohyoid phospho-ERK 1/2 content (mean ± SEM) expressed as normalized relative luminescence units; *n* = 5–7 per group; ^*^p < 0.05 and ^**^p < 0.01 vs. control, Tukey's multiple comparison test; Ctrl, normoxic control; Hypoxia, sustained hypoxia (FiO_2_ = 0.1); Tempol, tempol + sustained hypoxia; NAC, NAC + sustained hypoxia; RLU, relative luminescence units.

There was no change in sternohyoid p-p38 content following 6 weeks of CH with or without antioxidant supplementation (Figure 6A). However, 6 weeks of CH significantly decreased sternohyoid p-JNK content compared to control (One-Way ANOVA (*p* < 0.01) followed by Tukey's multiple comparison test (*p* < 0.05); chronic antioxidant supplementation with either tempol or NAC did not prevent this (Figure 6B). A similar effect was observed for sternohyoid p-ERK1/2 content. A Kruskal-Wallis test revealed a significant difference in the mean values of the groups. However, Dunn's multiple comparisons test revealed no significant differences between pairs (Figure 6C).

### Sternohyoid protein carbonyl and free thiol content after antioxidant treatment

**Figure 7:**
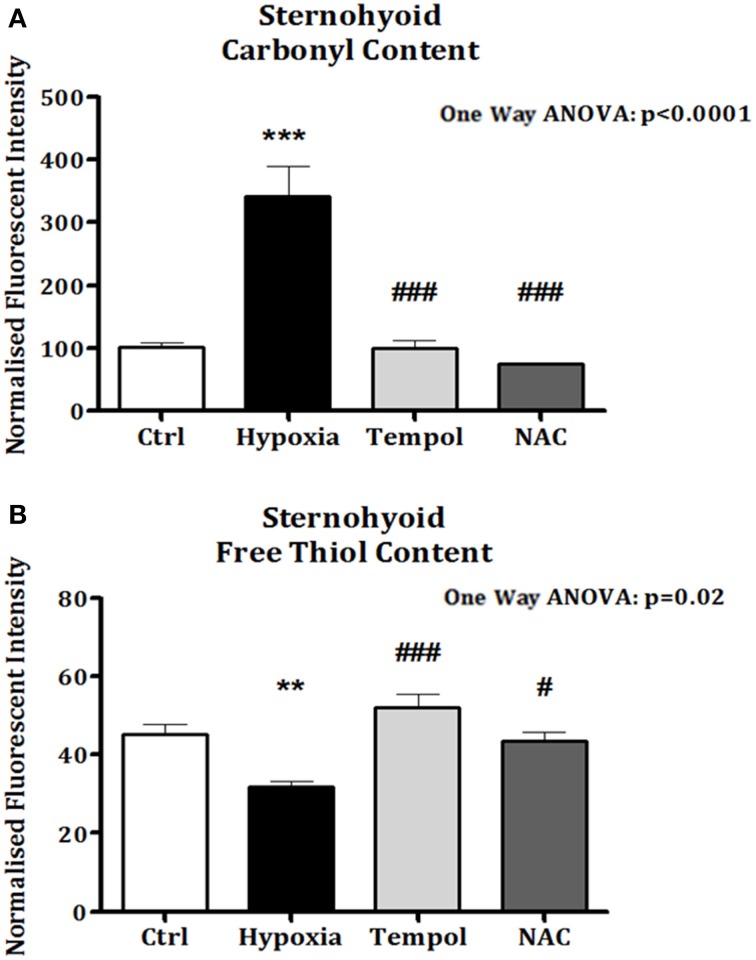
**Sternohyoid protein carbonyl and free thiol content following 6 weeks of normoxia and sustained hypoxia ± chronic antioxidant supplementation. (A)** Sternohyoid protein carbonyl content (mean ± SEM) expressed as normalized fluorescence intensity; *n* = 5–7 per group; **(B)** Sternohyoid protein free thiol content (mean ± SEM) expressed as normalized fluorescence intensity; *n* = 5–7 per group; ^**^p < 0.05 and ^***^p < 0.001 vs. control, ^#^*p* < 0.05 and ^###^ *p* < 0.001 vs. hypoxia, Tukey's multiple comparison test; Ctrl, normoxic control; Hypoxia, sustained hypoxia (FiO_2_ = 0.1); Tempol, tempol + sustained hypoxia; NAC, NAC + sustained hypoxia.

In the second cohort of animals, significant increases in sternohyoid protein carbonyl content were again observed after 6 weeks of CH (*p* < 0.001). Chronic antioxidant supplementation with either Tempol (*p* < 0.001) or NAC (*p* < 0.001) significantly ameliorated the CH-induced increase in protein carbonyl content (Figure 7A). Similarly, sternohyoid protein free thiol content was significantly lower than control after 6 weeks of CH (*p* < 0.01) and antioxidant treatment with Tempol (*p* < 0.001) and NAC (*p* < 0.05) ameliorated this effect (Figure 7B).

### Sternohyoid muscle function

There are no significant differences observed across groups for contractile kinetics, twitch force, V*max*, W*max*, or isotonic fatigue index (Table 3) as determined by One-Way ANOVA or Kruskal-Wallis test; however, CH exposure increased the contraction time, and decreased endurance (Table 3). NAC supplementation in CH increased peak twitch force, V*max*, W*max*, and endurance (Table 3). Fmax is significantly different across groups; decreased in CH compared to control and significantly increased by NAC supplementation in CH compared to CH alone as revealed by Tukey's multiple comparison *post-hoc* test (Table 3).

**Table 3:** **Sternohyoid muscle contractile properties after 6 weeks of normoxia and sustained hypoxia ± chronic antioxidant supplementation**.

	**Normoxia**	**Hypoxia**	**Tempol**	**NAC**	***P*-Value^a^**
TTP (ms)	9 ± 0	10 ± 0	11 ± 1	10 ± 0	ns
T50 (ms)	11 ± 1	11 ± 1	14 ± 3	12 ± 2	ns
Pt (N/cm^2^)	2.5 ± 0.4	1.6 ± 0.2	1.9 ± 0.2	3.0 ± 0.9	ns
F*max* (N/cm^2^)	12.3 ± 1.5	8.0 ± 0.6^*^	11.0 ± 0.3	16.0 ± 2.3^##^	<0.01
V*max* (Lo/s)	5.4 ± 0.7	5.2 ± 0.9	6.1 ± 0.7	6.1 ± 0.4	ns
W*max* (J/cm^3^)	0.6 ± 0.1	0.6 ± 0.1	0.8 ± 0.1	0.9 ± 0.1	ns
Fatigue Index (% of initial power)	63 ± 14	36 ± 7	33 ± 11	46 ± 12	ns

Data are presented as mean ± SEM; n = 5–8 per group;

a *One Way ANOVA*,

* p < 0.05 vs. Normoxia and

## *p 0.01 vs. Hypoxia, Tukey's post-hoc multiple comparison test; ns, not significant; Hypoxia, 6 weeks of sustained hypoxia (FiO_2_ = 0.1); Tempol, tempol + hypoxia; NAC, NAC + hypoxia*.

**Figure 8:**
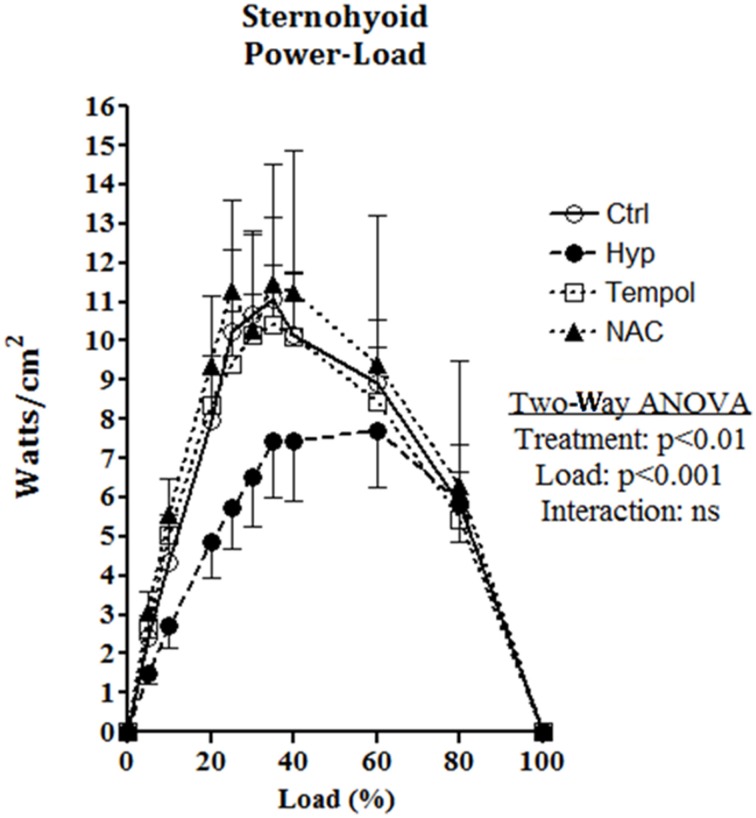
**Sternohyoid power-load relationship following 6 weeks of normoxia and sustained hypoxia ± chronic antioxidant supplementation**. Sternohyoid specific power (mean ± SEM) expressed as Watts/CSA (cm^2^) as a function of load expressed as a percentage of peak force (force/peak force^*^100); *n* = 5–7 per group; Ctrl, normoxic control; Hyp, sustained hypoxia (FiO_2_ = 0.1); Tempol, tempol + sustained hypoxia; NAC, NAC + sustained hypoxia. *P*-values shown for two factor (treatment × load) analysis of variance highlighting a significant effect of 6 weeks of sustained hypoxia on sternohyoid power-load relationship. Antioxidant supplementation reversed the effects of hypoxia.

**Figure 9:**
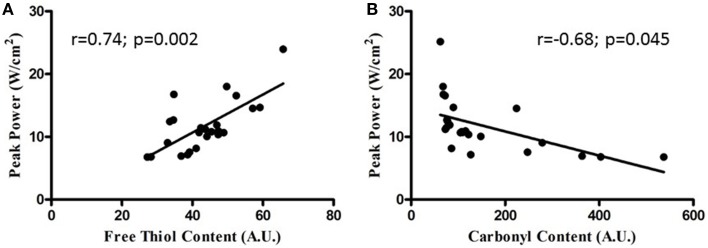
**Relationship between sternohyoid power and oxidative stress markers**. Relationship between sternohyoid specific peak power and muscle oxidative stress markers [protein free thiol content **(A)** and carbonyl content **(B)**] plotting data from all animals in the second cohort of studies; *n* = 24. Note the correlation coefficients and statistically significant relationships.

Sternohyoid muscle isotonic contractile function (power) was significantly depressed by CH exposure (Figure 8; Two-Way ANOVA: gas treatment: *p* < 0.001). Significant correlations were observed for oxidative stress markers and sternohyoid muscle function (Figure 9). The deleterious effects of CH exposure on sternohyoid function were prevented with antioxidant supplementation in CH using either tempol or NAC (Figure 8).

## Discussion

This is the first study to identify proteins in an upper airway muscle undergoing redox remodeling following exposure to CH. CH decreased sternohyoid force- and power-generating capacity. Antioxidant supplementation prevented CH-induced sternohyoid dysfunction. The main findings of this study are: (1) CH induces temporal changes in protein carbonylation and free thiol oxidation in the sternohyoid, responses different to that seen in limb muscles; (2) Proteins key to muscle contraction, metabolism, and homeostasis are redox-modified in the sternohyoid following 6 weeks of CH; (3) Glycolytic enzyme activities are temporally affected in the sternohyoid following progressive CH exposure; (4) CH has no effect on sternohyoid HIF-1α content; (5) CH decreases p-JNK in sternohyoid independent of antioxidant status; (6) CH causes sternohyoid muscle dysfunction; (7) Oxidative stress markers and muscle function were significantly correlated; (8) Both protein oxidation and contractile impairment following CH are fully reversed by chronic antioxidant supplementation.

### Muscle redox status

In skeletal muscle adaptation to high altitude and respiratory diseases featuring hypoxia, ROS have been shown to cause protein redox remodeling and to modify protein function with resultant changes in metabolic processes, homeostasis and contractile performance (Zuo and Clanton, 2005; Marin-Corral et al., 2009; Murray, 2009; Chaudhary et al., 2012; Levine et al., 2013; Puig-Vilanova et al., 2015). However, the cause and motive for increased ROS production is unclear and may differ in skeletal muscles given their varied functional roles. In the present study, we observed time-dependent increases in protein oxidation (both increased carbonyl content and decreased free thiol content) in CH-exposed sternohyoid muscle (differential to responses in limb muscles, which is likely pivotal for time-dependent and differential structural and functional remodeling reported in previous studies (El-Khoury et al., 2003, 2012; Faucher et al., 2005; Gamboa and Andrade, 2010, 2012; McMorrow et al., 2011; Carberry et al., 2014). This, in turn, may be of clinical significance given that CH is potentially implicated in the progression of respiratory diseases.

Clearly there is differential muscle protein redox remodeling which appears unrelated to fiber type *per se* given the similarities between the EDL and sternohyoid muscles, and unrelated to respiratory vs. limb muscle differences *per se* given the differences observed in the EDL compared with the soleus. As the soleus is a postural muscle, it exhibits greater contractile activity than the EDL which is used for more powerful movements of the limbs. The combination of activity and hypoxic exposure is potentially synergistic in driving protein oxidation, with reflex hyperventilation and hypoxia combining to drive protein oxidation in the sternohyoid. Furthermore, a comparison of basal carbonyl signals from sternohyoid and limb muscle proteomes suggests that basal workload of a given muscle is more important in determining carbonylation of proteins than fiber type *per se*.

2D redox proteomic profiling of the sternohyoid muscle after 6 weeks of CH exposure reveals key proteins in muscle that are targeted by ROS, many of which are redox modified in COPD muscle (Marin-Corral et al., 2009). It is clear from Table 2 that extensive remodeling occurs to mitochondrial, metabolic, and associated proteins, suggesting that the mitochondria are the primary source of remodeling stress. Reflex hyperventilation increases metabolic demand and subsequent metabolic substrate flux to the respiratory chain. With hypoxia, stockpiling of electrons occurs, increasing electron leak and subsequent ROS formation. This is supported by the findings of decreased muscle mitochondrial density and mitochondrial remodeling in hypoxia, high altitude, and respiratory-related diseases (Hoppeler et al., 1990, 2003; Gosker et al., 2000; Wijnhoven et al., 2006; Viganò et al., 2008; Gamboa and Andrade, 2010, 2012). The fold change in free thiol content following 1 week of CH is greatest in soleus>EDL>sternohyoid suggesting that fiber type, more so than contractile activity, is key to this change.

EDL free thiol content remains elevated above control whereas carbonyl content was unchanged after 6 weeks of CH; however the pattern of change is similar to the sternohyoid and soleus muscles. It is likely to be a combination of hypoxia, muscle fiber composition, and workload in hypoxia that accounts for the differential changes in respiratory and limb muscle protein oxidation and functional changes. In keeping with the notion of muscle specific effects of CH, other studies have reported differential effects of oxidation, based on carbonyl and thiol measurements as well as other indicators of altered cellular oxidative/reductive state which are proposed to depend on the activity and/or local cellular environment of the relevant protein (Haycock et al., 1998; Kadiiska et al., 2005; El-Shafey et al., 2011). See additional discussion in the supplementary file.

### Muscle redox signaling

Decreased sternohyoid GAPDH activity with progressive hypoxia is consistent with the decreased expression measured by proteomic profiling after 6 weeks of CH. CH-induced changes in LDH activity in the sternohyoid follow a similar pattern to GAPDH. As muscle function and metabolism are intrinsically interlinked through HIF signaling (Semenza et al., 1994; Vogt et al., 2001; Kim et al., 2006), one might have expected a CH-induced increase in HIF-1α content. However, decreased GAPDH expression and decreased GAPDH and LDH activities observed in the sternohyoid muscle of our mouse model are suggestive of decreased HIF-1α content. Interestingly, after 6 weeks of CH exposure we observed that there was no change in sternohyoid HIF-1α content. It is plausible that the degree of hypoxia experienced by the sternohyoid may change temporally with acclimatization to hypoxia such that HIF-1α may have been activated earlier in the exposure. Of note, for example, GAPDH activity is increased in sternohyoid muscle after 1 but not 3 weeks of CH. Protein carbonylation is elevated in CH sternohyoid after 6 weeks suggesting that although ROS are presumably elevated in the muscle there is no ROS-dependent HIF-1α activation. Moreover, antioxidant supplementation did not affect HIF-1α content in CH sternohyoid. Thus, our results suggest that redox modulation of sternohyoid metabolism and function is HIF-1α independent.

The MAPK proteins are involved in a diverse array of cellular signaling events and are vital to muscle growth and homeostasis. p38 activity in muscle is strongly associated with promoting atrophy in COPD limb muscle (Lemire et al., 2012) and is potentially required for hypoxia signaling of HIF via mitochondrial ROS production (Emerling et al., 2005). However, neither HIF-1α content nor chymotrypsin-like 20S proteasome activity changed in the sternohyoid muscle following CH. Indeed, hypertrophy of fast fibers in the sternohyoid is reported following CH exposure in the rat (McMorrow et al., 2011) suggesting enhanced protein synthesis. This is especially interesting, as the present study has established for the first time, that the CH sternohyoid has decreased (not increased) power-generating capacity, highlighting a potent redox-dependent inhibitory effect of CH on the contractile machinery of the airway dilator muscle, rather than CH-induced muscle atrophy.

p-JNK content is decreased in the sternohyoid following CH exposure. Differential regulation of p38 and JNK occurs at the MAP2K level and is potentially influenced by contractile activity and mechanical stress (Sawada et al., 2001). The combination of hypoxic/redox stress, muscle fiber type combination, and mechanical activity of the sternohyoid is likely important in determining the stress kinase response to CH exposure. A ROS/p-JNK-dependent mechanism is implicated in CH-induced sternohyoid dysfunction but it is important to note that antioxidant supplementation did not prevent the CH-induced decrease in p-JNK (or p-ERK1/2), whereas antioxidants prevented sternohyoid redox stress and contractile dysfunction following CH exposure.

### Sternohyoid isotonic performance

The sternohyoid force- and power-generating capacity was significantly depressed by CH exposure. Muscle oxidative stress markers and contractile power were significantly correlated; moreover, antioxidant supplementation during CH exposure prevented both protein redox stress and contractile dysfunction. Therefore, we posit that CH-induced redox remodeling of key proteins in metabolism and the contractile apparatus is central to CH-induced sternohyoid muscle dysfunction. Concerning the potential clinical relevance of our findings, sternohyoid dysfunction could serve to increase obstructive airway events *in vivo*—further disrupting respiratory homeostasis in diseases characterized by hypoxic stress. We speculate that CH-induced redox remodeling and functional deficit in pharyngeal dilator muscles potentially underpins the higher prevalence of OSA observed in COPD patients (the overlap syndrome) (Owens and Malhotra, 2010).

## Summary and conclusions

This study highlights the application of a redox proteomics approach to a translational animal model of hypoxia. CH exposure causes progressive redox remodeling in the sternohyoid (upper airway dilator) muscle with differential responses to limb muscles. CH results in metabolic protein structural remodeling with resultant changes in enzyme activity. Redox and expression changes were shown to occur to various protein chaperones including those important to cross-bridge maintenance. Ultimately, it appears that CH-induced redox changes result in functional deficit in the sternohyoid, given that antioxidant supplementation during CH exposure prevents both protein stress and functional impairment. We hypothesize a pivotal role for ROS in pharyngeal dilator muscle structural and functional (mal)adaptations to CH. The putative role of hypoxia in driving respiratory muscle remodeling in human respiratory disease warrants investigation.

## Author contributions

The study was conceived and designed by PL and KO. Experiments and data analyses were performed by PL in the laboratories of KO and DS. Mass spectrometry experiments were performed and analyzed by RS in the laboratory of AV. The manuscript was written by PL and KO with contributions from the other authors.

### Conflict of interest statement

The authors declare that the research was conducted in the absence of any commercial or financial relationships that could be construed as a potential conflict of interest.
