# Supplementary material for: Chronic sustained hypoxia-induced redox remodeling causes contractile dysfunction in mouse sternohyoid muscle
Source: Front Physiol. 2015 Apr 20;6:122. doi: 10.3389/fphys.2015.00122 (PMC4403307; doi:10.3389/fphys.2015.00122)
Supplement: Supplementary file 1 [file SupplementaryDiscussion.DOCX]

# Supplementary discussion

# Remodeled sternohyoid proteins following CH as identified by 2D redox proteomics and mass spectrometry

2-oxo-glutarate dehydrogenase, glycogen phosphorylase, and carbonic anhydrase III (CAIII) present with greatest remodeling in this study. Redox-remodeling of 2-oxo-glutarate dehydrogenase, a TCA cycle enzyme, is likely to limit ROS production by both its own oxidoreductase activity and by mitochondria (Tretter and Adam-Vizi, 2005). CAIII rehydrates CO_2_; a potential role for this enzyme in lactate shuttling through monocarboxylate transporters and other transporters has also been suggested (Becker *et al*, 2014). Interestingly, redox remodeling of this protein is also observed in respiratory muscle of COPD patients (Marin-Corral *et al*, 2009) and CAIII has been shown to be significantly up-regulated after exercise in humans but only in hypoxia (Zoll *et al*, 2006). Furthermore, CAIII has been shown to have important anti-apoptotic effects in oxidatively stressed cells (Räisänen *et al*, 1999) and may confer protection for the upper airway dilator muscle in hypoxia both as an antioxidant and pH regulator. Glycogen phosphorylase regulates glycogen breakdown, and is increased more than 4-fold after CH exposure in the sternohyoid. Of interest, no change is observed for this enzyme in human vastus lateralis with ascent to high altitude (Young *et al*, 1984), suggesting that it may be of selective importance to the sternohyoid. The large up-regulation would be expected to increase carbohydrate substrate availability for energy production, required to maintain the increased continuous rhythmic function in the sternohyoid during CH-induced hyperactivity. Furthermore, the ATP-producing capacity of the “second” part of the glycolytic pathway may be enhanced through the increased expression of phosphoglycerate kinase, phosphoglycerate mutase and the pyruvate kinase isozymes. This enhanced step 2 of glycolysis, alongside increased glycogen phosphorylase content, is potentially fundamental to the maintenance of enhanced sternohyoid contractile activity in hypoxia.

Creatine kinase, aldolase, and aconitase are metabolic proteins susceptible to oxidation and this is confirmed in the present study in the sternohyoid muscle after 6 weeks of CH exposure. Oxidation of these enzymes correlates with negative regulation of their activities (Barreiro *et al*, 2005; Marin-Corral *et al*, 2009). Aconitase is often measured as a marker of oxidative stress. Creatine kinase deficiency is associated with deficiencies in burst activity of muscle (van Deursen *et al*, 1993) and this may have particular relevance to the sternohyoid from a functional perspective as burst-like activity during recruitment is required to reopen the airway when occlusions occur during sleep (Remmers *et al*, 1978).

Carbonylation of α-crystallin B chain, a cross-bridge chaperone described as essential to myofilament maintenance (Elliott *et al*, 2013), is observed. Forms of CRYAB gene-linked myopathy can be suppressed by altering availability of reducing equivalents such as NADPH, supporting a role for ROS regulation (Xie *et al*, 2013). Furthermore, α-crystallin B chain is described as having antioxidant-like properties (Manzanares *et al*, 2001). Of note, strength training increases its expression (Paulsen *et al*, 2012). Just as the maintenance of proper folding of cross-bridge proteins in working muscle is fundamental to function, α-crystallin B chain may be an important player in the differential functional adaptations observed in respiratory and limb muscles (El-Khoury *et al*, 2003, 2012; McMorrow *et al*, 2011; Gamboa and Andrade, 2012; Carberry *et al*, 2014).

Myoglobin is an oxygen binding protein with a ferrous core that is expressed in skeletal muscle. Myoglobin presents with decreased expression in the CH sternohyoid which may be important in reducing the formation of the extremely reactive hydroxyl radical by decreasing Fenton reactions. Decreased myoglobin expression has been previously observed in skeletal muscle in response to hypoxia (Kanatous *et al*, 2009).

Heat-shock proteins (HSPs) are correlated with skeletal muscle fiber type (Locke *et al*, 1991) and are involved in maintaining mitochondrial integrity (Young *et al*, 2003), so their respective modulation may have a role in differential functional outcomes in respiratory and limb muscles. Small HSPs have been shown to modulate ROS, raise GSH levels, prevent apoptosis signaling derived from stressed mitochondria, and activate the proteasome (Préville *et al*, 1999; Aiken *et al*, 2011; Christopher *et al*, 2014) while it is also plausible that oxidative modification and/or expression changes of protein chaperones facilitates atrophy instead of re-folding (Kästle *et al*, 2012).

While GAPDH expression is decreased in the sternohyoid after CH, reductive redox remodeling is observed similar to glycogen phosphorylase which suggests lesser oxidation, protection, or both increased translation and ROS-induced degradation occurring simultaneously albeit at different rates. The bi-phasic change in sternohyoid GAPDH activity with progressive hypoxia combined with the temporal changes in total protein carbonyl and free thiol content could conceivably be further suggestive of ROS-dependent phenomena. GAPDH is a key metabolic switch protein. With oxidation, GAPDH relocates to the nucleus (Hwang *et al*, 2009) and metabolic substrate flux is directed toward the pentose phosphate pathway to increase the reducing power of the cell by NAD(P)H formation.
